# The Prevalence of Thrice, Twice, and Once Human Papillomavirus DNA Positivity in Older Chinese Women

**DOI:** 10.3389/fmed.2020.00391

**Published:** 2020-09-02

**Authors:** Guomin Hu, Jun Xu, Ling Xu, Longmei Jin

**Affiliations:** ^1^Department of Obstetrics and Gynecology, Minhang Hospital, Fudan University, Shanghai, China; ^2^Minhang District Maternal and Child Health Hospital, Shanghai, China

**Keywords:** human papillomavirus (HPV), repeated HPV DNA detection, community population, older women, negative for intraepithelial lesion or malignancy (NILM)

## Abstract

**Background:** Human papillomavirus (HPV) DNA detection in cervical samples is widely used to identify HPV infection; however, there is little detailed evaluation of the characteristics of HPV prevalence by repeated DNA detection in community populations.

**Methods:** Beginning in 2014, a Papanicolaou (Pap) smear and HPV cotesting program was implemented among older women living in the Minhang district of Shanghai. This report uses information from 225,000 participants, who provided person-time data. Of these, 632 subjects had 3 repeated visits and at least one HPV DNA-positive result in the last 5 years.

**Results:** All 16 genotypes of HPV displayed thrice, twice, and once positivity results in 3 repeated tests and differed by proportions among and within genotypes. HPV52 and 58 are the two most dominant genotypes in total and in thrice positive person-time. The thrice positive person-time exceeded 50% in each of HPV58-, 35-, 52-, 56-, 18-, 68-, 31-, and 16-infected women. The single positive person-time ratio ranged from 7.9% (HPV35) to 38.9% (HPV11). Age differed among and within genotypes in thrice, twice, and once positive women. The average age of HPV-free controls was 59.0 ± 7.2 yo, which is close to the median of average ages for thrice and twice positive women and is older than most average ages for once positive women. The percentages of negative results for intraepithelial lesion or malignancy (NILM) for thrice HPV52-, 58-, 16-, 56-, and 59-positive women were significantly lower than the percentage of NILM for HPV-free women.

**Conclusion:** Thrice and/or twice HPV DNA positivity are common in HPV-infected women and tend to occur in older women.

## Introduction

It has been established that persistent high-risk human papillomavirus (HR-HPV) infection is associated with high-grade cervical precancer (1–3). In theory, a persistent or transient infection caused by a given virus is determined primarily by the organ or cells it infects and the host's immune response. HPVs infect basal proliferating keratinocytes but express high levels of viral proteins and replicate only in the upper layers of the stratum spinosum and granulosum of the squamous epithelia (4, 5). Since keratinocytes undergo a specialized form of programmed cell death, cornification (6), the natural history of HPV infection should be influenced by the apoptosis of virus-laden keratinocytes (5). In addition, viral genome replication, viral protein expression, and virus assembly and release all occur only at the basal layer of the epithelium, which is an interface zone for the host's innate and adaptive immunity (3–5). These characteristics determine the particularity of the natural history of HPV infection. However, studies suggest that cell-mediated immunity is a main force that repels most HPV infections (3, 5), and the time required for clearance of the HR-HPV types (viral DNA can no longer be detected) averages 8–14 months (7–9).

HPV persistence is commonly defined as two or more viral DNA-positive time points (7–11). A recent meta-analysis showed that the average median duration of any HPV infection was 9.8 months (11), outlining that HR-HPV infection had a slightly longer median duration of 9.3 months than that of low-risk HPV (LR-HPV), which is 8.4 months (11). In terms of persistent duration, the top three HPVs were HPV31, HPV33 and HPV16, with weighted averages of 14.4, 12.5, and 12.4 months, respectively (11). However, there is still no reasonable uniform definition of persistent infection in clinical practice, and current estimates of persistence vary greatly and differ by patient age, HPV genotype, detection method, treatment, and HPV testing interval (12).

Learning how long an HPV infection will persist has practical significance. Although persistent infection is theoretically determined primarily by the organ HPV infects and the host's immune response, the exact natural history of HPV infection is difficult to reveal owing to the lack of classic dynamic immune processes shown by a virus that causes viremia. Thus, repeated HPV DNA detection could promote the understanding of the duration of virus infection. Under this premise, a study focused on infection persistence is critical in determining the temporal correlation between a given HPV infection and the occurrence of cervical lesions. In addition, an accurate understanding of the duration of virus infection provides a theoretical basis for improving the ability to clear HPV infection by procedures such as loop excision, conization, and cryotherapy.

Beginning in 2014, a Pap smear and HPV cotesting program was implemented among older women living in the Minhang district of Shanghai. Among person-time data from 225,000 participants, 632 subjects had 3 repeated visits and at least one HPV DNA-positive result in the last 5 years. This report evaluates the proportions, age distribution, and cervical cytological examination of thrice, twice, and once HPV DNA-positive women.

## Methods

### Study Design and Participants

This is a retrospective study focused on older women living in the Minhang district of Shanghai. As we reported previously (2), the Shanghai Minhang District Health and Family Planning Commission have implemented a community-based Pap smear and HPV cotesting screening program in the Minhang district, which has a population of 2.43 million in nine towns. Commencing in 2014, screening was implemented in four towns with more than 50,000 women screened annually, some of whom took part in multiple annual screenings from 2014 to 2019. In general, the interval of repeated visits is 12 months. The visit date, HPV results, and cytopathological results of these participants are recorded annually. The results outlined in this report involved participants who underwent cotesting 3 times and had ≥1 positive HPV DNA result for any HPV strain.

### Cytological Examinations

As we reported previously (2), all cytological examination procedures strictly followed the 2014 Bethesda System for Reporting Cervical Cytology (13). Each cervical sample for cytological examination was taken using a sterile cytobrush inserted into the cervical os and rotated 360°. The cells were then evaluated as recommended by the manufacturer of the BD SurePath liquid-based Pap test (Becton, Dickinson and Company, Franklin Lakes, NJ, USA). The results, cervicitis/no intraepithelial lesion or malignancy (NILM); atypical squamous cells—of undetermined significance (ASC-US); atypical squamous cells—cannot exclude high-grade squamous intraepithelial lesion (HSIL) (ASC-H); low-grade squamous intraepithelial lesion (LSIL); and HSIL, were reported using the Bethesda System for Reporting Cervical Cytology; Definitions, Criteria, and Explanatory Notes; Third Edition (13). Those with ASC-US and higher cytological grades underwent colposcopy and biopsy; four lesion-directed biopsy samples were obtained from distinct epithelial regions in the cervical transformation zone, which turned white upon application of 5% (v/v) acetic acid. All biopsies were ranked in order of lesional severity at the time of colposcopy and were evaluated by at least two certified pathologists blinded to the clinical data.

### HPV Genotyping

As we reported previously (2), at the time of Pap testing, cervical specimens for HPV testing were collected using a sterile cytobrush as described above, and viral DNA was extracted using QIAamp DNA Mini Kits (QIAGEN, Shanghai, China). HPV DNA evaluation and genotyping were performed using a kit provided by Huada Biotech Co., Ltd. (Wuhan, China). The kit detects 12 HR-HPVs (16, 18, 31, 33, 35, 39, 45, 51, 52, 56, 58, and 59), two possibly carcinogenic genotypes (66 and 68), and two LR-HPV strains (6 and 11). The kit employs polymerase chain reaction followed by HPV genotype-specific DNA microarray analysis. It has been approved by the Chinese Food and Drug Administration. All procedures followed the manufacturer's protocols.

### Statistical Analysis

Because our participants had 1–3 results for a given variable, person-time was adopted in the statistical analysis. Continuous variables are presented as the means ± standard deviations, and categorical data are presented as frequencies with percentages. Differences between and within groups were evaluated using one-way ANOVA, *t*-test, chi-squared test, or Fisher's exact probability test, as appropriate. All statistical analyses were performed with the aid of SPSS software (ver. 13.0; SPSS Inc., Chicago, IL, USA), and the significance level was set at α = 0.05.

## Results

### A General Description of Participants

**Figure 1:**
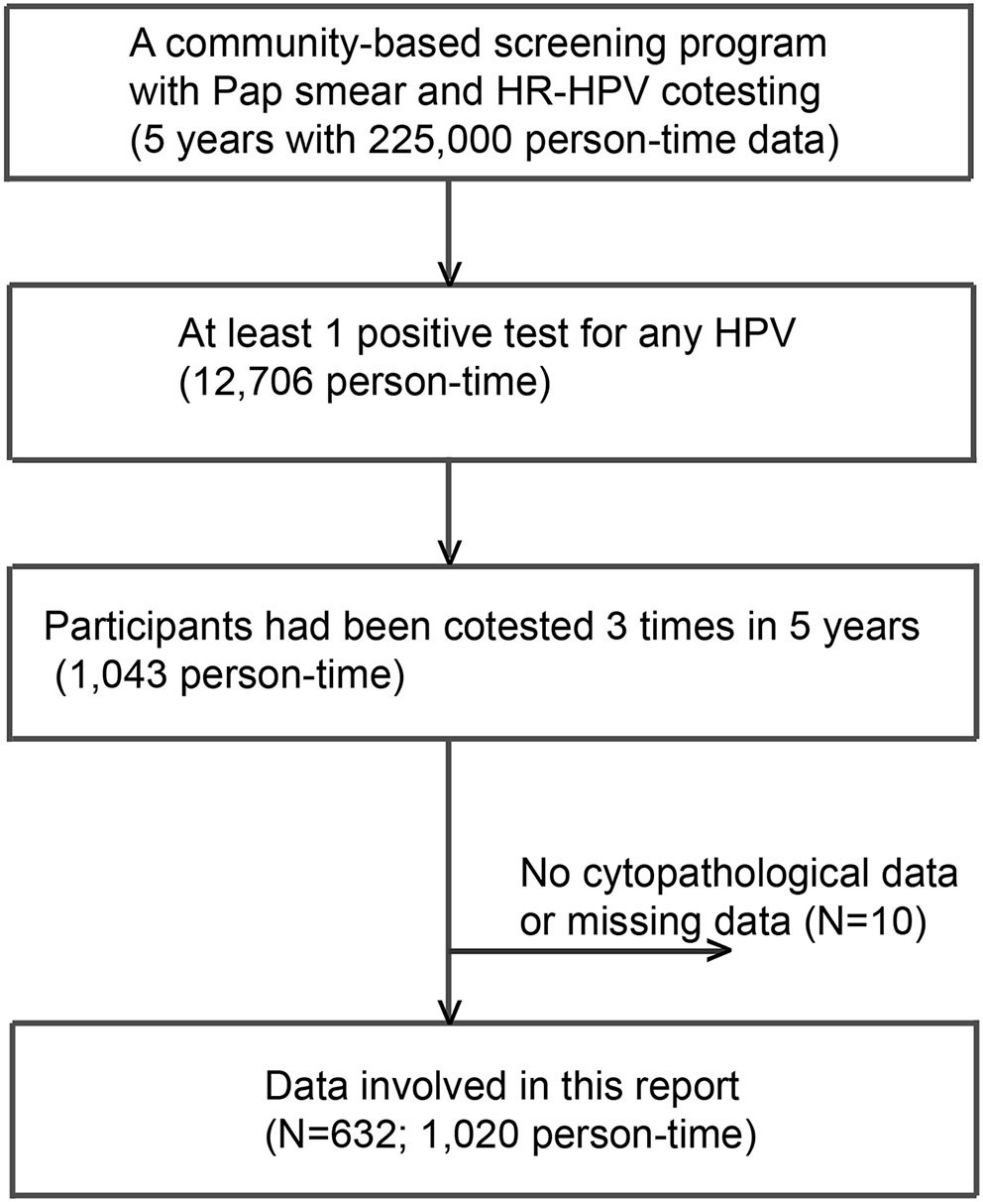
A flowchart of participants included. A total of 632 subjects with 1,020 person-time data sets were involved in this analysis.

A community-based screening program with Pap smear and HPV cotesting yielded 225,000 person-time data from 2014 to 2019. A total of 12,706 person-times had at least 1 positive test for any HPV DNA over the 5 years. Among these, 632 subjects with 1,020 person-time data were involved in this analysis (Figure 1). All HPV-negative subjects (*N* = 23,196) assembled in the first half of 2017 were adopted as “controls.” As shown in Table 1, the numbers of subjects with thrice, twice, and once positive results for a given HPV DNA were 269, 131, and 231, respectively. A total of 95.2% of HPV-infected women and 97.8% of HPV-free women showed cytological results of NILM (Table 1).

**Table 1:** A general description of the subjects.

	**HPV infected**	**HPV free**
Number of subjects	632	23,196
Person-years	1020	23,196
Age, yo	57.0 ± 6.8	59.0 ± 7.2
Number of subjects thrice positive for one HPV strain	269 (42.6%)	0
Number of subjects twice positive for one HPV strain	132 (20.9%)	0
Number of subjects once positive for one HPV strain	231 (36.6%)	0
NILM, person-years	971 (95.2%)	22,683 (97.8%)
LSIL, person-years	2 (0.2%)	3 (0.01%)
HSIL, person-years	2 (0.2%)	0 (0.0%)
ASC-US, person-years	45 (4.4%)	509 (2.2%)

*NILM, negative for intraepithelial lesion or malignancy; LSIL, low-grade squamous intraepithelial lesion; HSIL, high-grade squamous intraepithelial lesion; ASC-US, atypical squamous cells–of undetermined significance*.

### Percentages of Thrice, Twice, and Once HPV DNA-Positive Person-Time Among Strains

**Figure 2:**
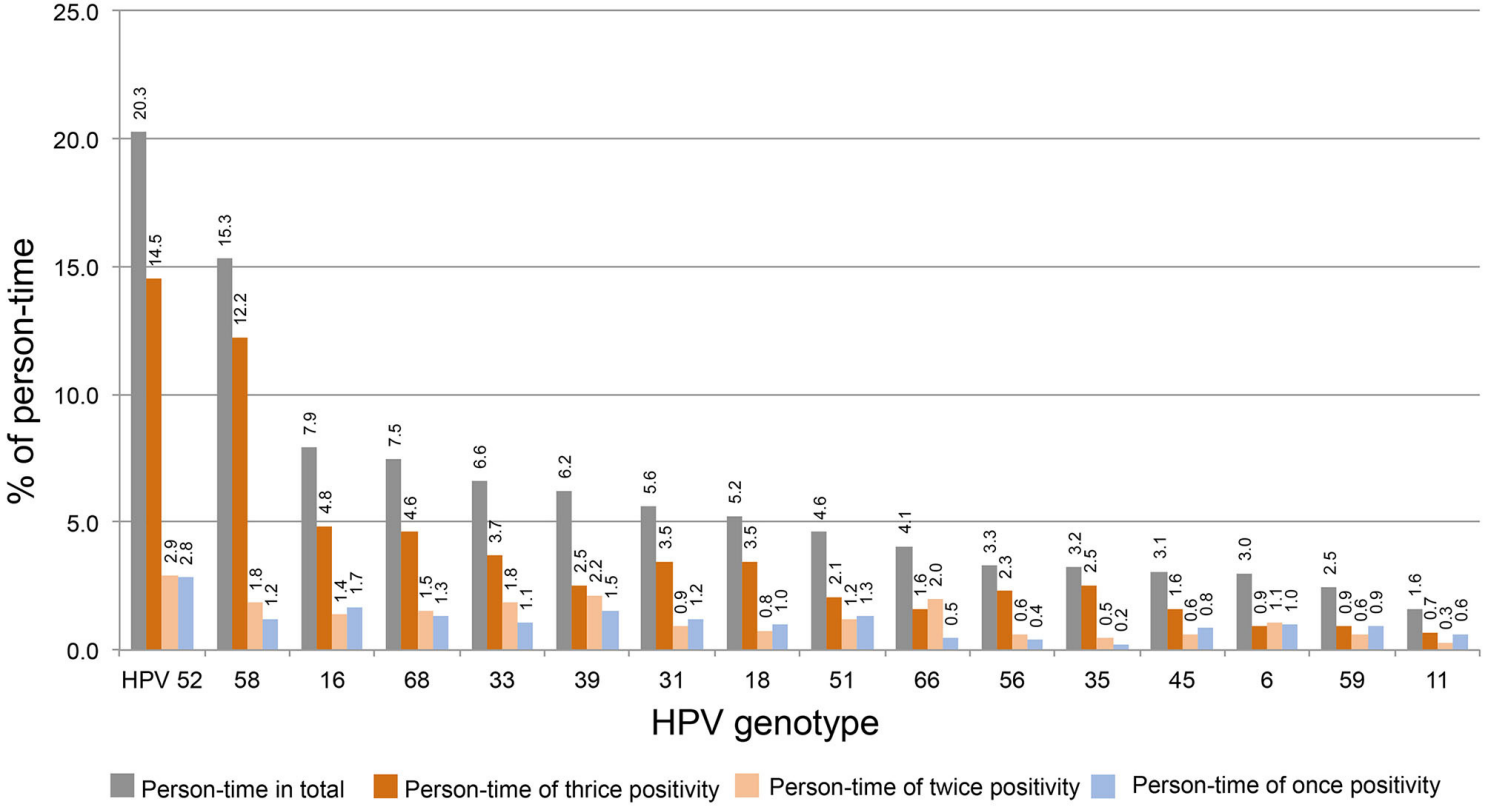
The distribution of thrice, twice, and once HPV DNA positivity among strains. The person-time of each HPV strain was for calculated thrice, twice, and once HPV DNA positivity. The percentage of above person-times was calculated by dividing the total person-time by 1,020 and plotted in order from high to low (thrice and twice positive).

The person-times of thrice, twice, and once HPV DNA-positive tests for each strain were calculated. To determine the distribution of thrice, twice, and once HPV DNA-positive rates among strains, the above person-times were divided by total person-time (1,020) and plotted in order from high to low thrice and twice positive rates. As shown in Figure 2, HPV52 is the dominant strain with the highest percentages of total positive (20.3%), thrice positive (14.5%), twice positive (2.9%), and once positive tests (2.8%). HPV58 is the 2nd most dominant strain, and the percentages of total and thrice HPV DNA-positive tests were 15.3 and 12.2%, respectively.

**Figure 3:**
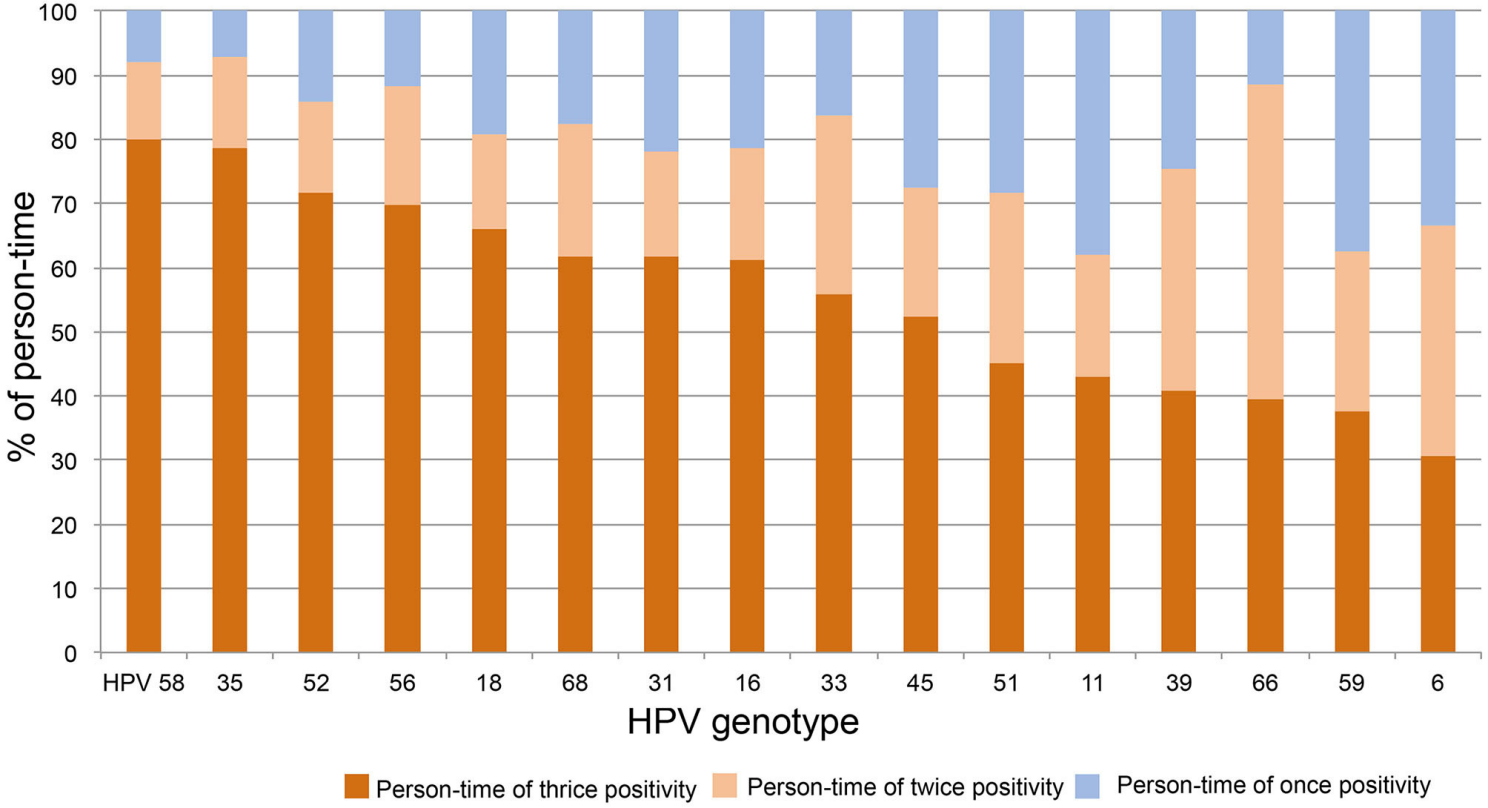
The distribution of thrice, twice, and once HPV DNA positivity within strains. The person-time of each HPV strain was calculated for thrice, twice, and once HPV DNA positivity. The percentage of above person-times was calculated by dividing the total person-time of each HPV strain and plotted in order from high to low for thrice DNA positivity.

To determine the distribution of thrice, twice, and once HPV DNA-positive rates within each strain, the above person-times were divided with the total person-time of each HPV strain and plotted in order from high to low of thrice positivity. As shown in Figure 3, the person-time of thrice HPV58, 35, 52, 56, 18, 68, 31, 16, 33, and 45 DNA-positive surpassed 50%. Together with the results of twice DNA positivity, the person-times of twice and thrice DNA positivity all exceeded 60%.

In this report the period duration (last test date – first test date) of thrice and twice HPV DNA positive tests ranged from 2 to 4 years. Since this study is not specially designed to determine the true duration of HPV infection and the time nodes are artificially controlled, we did not pursue further analysis of the period here.

### Age Difference Among and Within HPV Strains

**Figure 4:**
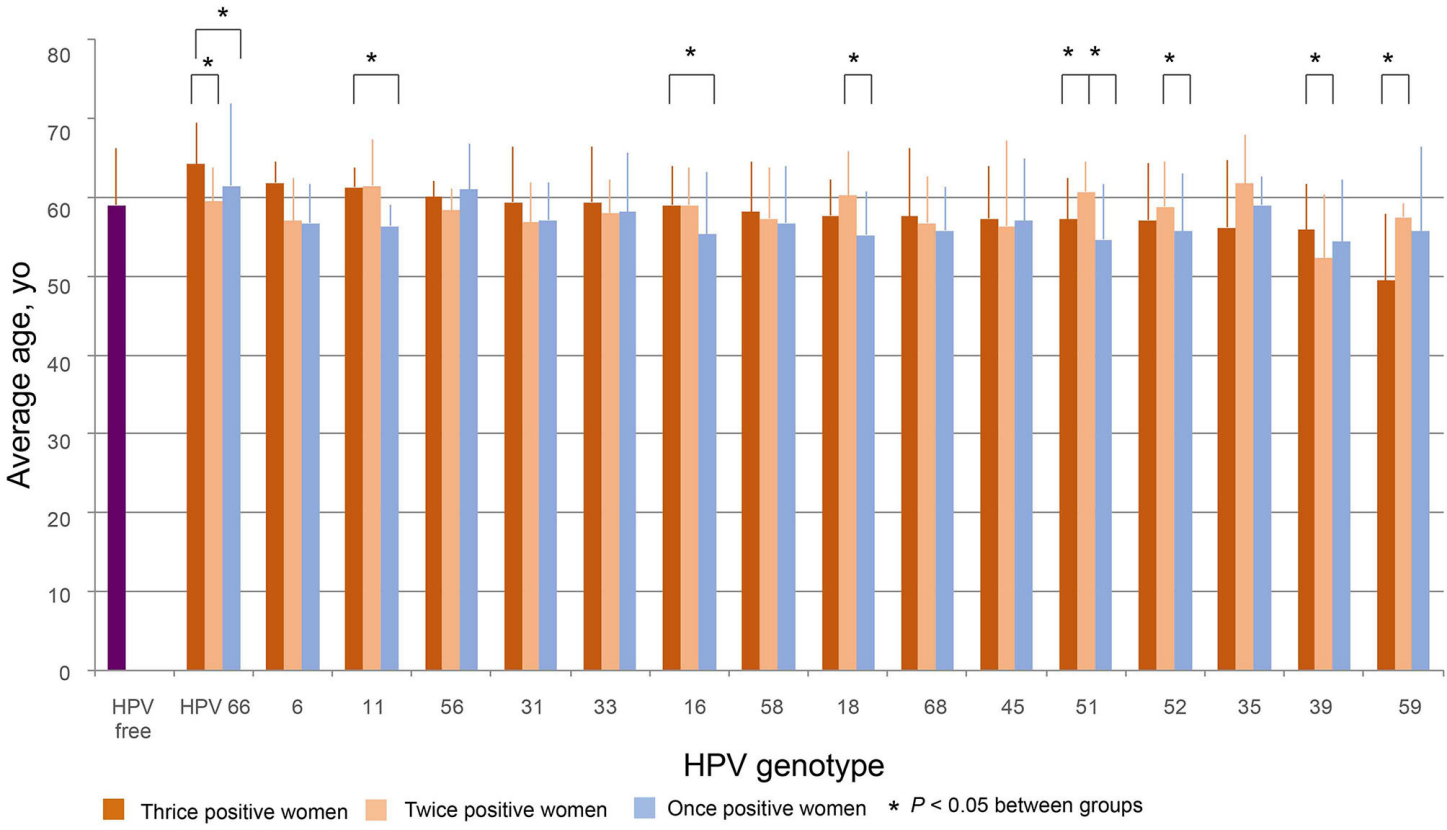
The distribution of age. The average ages of women thrice, twice, and once positive for HPV DNA were calculated by strain. The figure was plotted in order from high to low according to the average ages of women with thrice HPV DNA-positive results. Differences among and within HPV strains were evaluated by *T*-tests.

The average ages of women thrice, twice, and once positive for HPV DNA were calculated for each strain. One-way ANOVA showed that the average ages differed by strain in both thrice and twice HPV DNA-positive women. Figure 4 was plotted by the average ages from high to low of thrice HPV DNA-positive women. The average age for women with thrice HPV66 DNA-positive results was 64.2 ± 5.3 yo and declined to 49.4 ± 8.6 yo for HPV59. The oldest and youngest average ages for twice HPV DNA-positive women were 61.7 ± 6.2 yo (HPV35) and 52.3 ± 8.0 yo (HPV39). The average ages for HPV DNA-positive women ranged from 54.4 ± 7.8 yo (HPV39) to 61.5 ± 10.5 yo (HPV66) among strains (Figure 4).

The average age of HPV-free controls was 59.0 ± 7.2 yo, which was close to the median of the average ages for thrice and twice HPV DNA-positive women and older than most of the average ages for once HPV-positive women (Figure 4).

For HPV66 and 39, the average ages of thrice positive women were significantly greater than those of twice positive women. For HPV66, 11, 16, and 51, the average ages of thrice positive women were significantly older than those of once positive women. For HPV18, 51, and 52, the average ages of twice positive women were significantly greater than those of once positive women. As an exception, for HPV59, the average ages of twice positive women were significantly greater than those of thrice positive women (Figure 4).

### Differences in NILM Distribution Among and Within Strains

**Figure 5:**
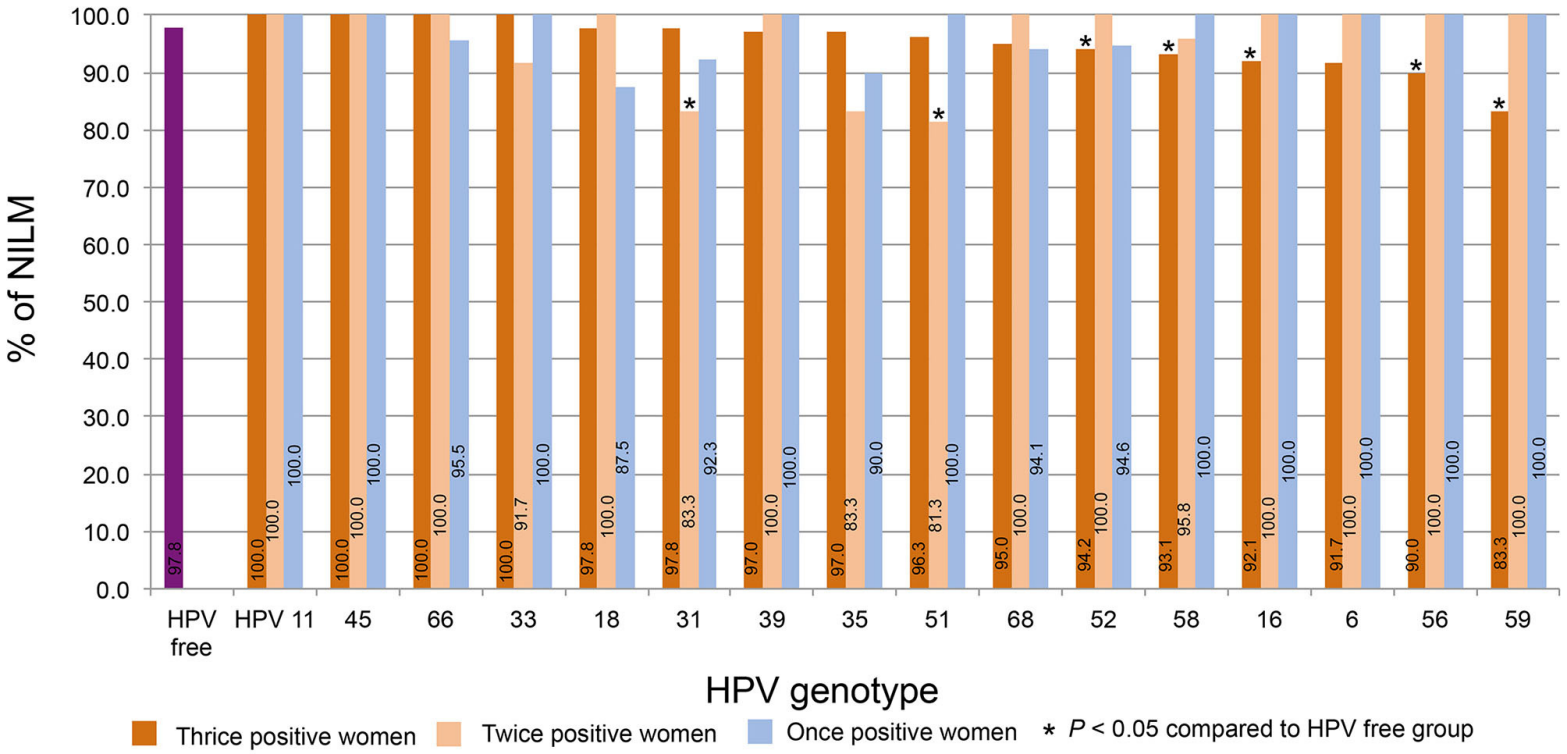
The distribution of NILM. The percentages of NILM cervical cytology results for women with thrice, twice, and once positivity for HPV DNA were calculated by strain. The percentages were plotted in order from high to low according to the percentages of women with thrice HPV DNA-positive results.

The percentages of NILM in women who were thrice, twice, and once positive for HPV DNA were calculated by strain. The percentage of women NILM among HPV-free women was 97.8%. For the thrice HPV DNA-positive women, Figure 5 shows the order from high to low according to the NILM percentages. The percentages for HPV11, 45, and 66 were 100% and declined to 83.3% for HPV59; the percentages for HPV52, 58, 16, 56, and 59 were significantly lower than the percentage of HPV-free women. Of twice HPV DNA-positive women, most HPV strain-infected women displayed 100% NILM, while the NILM percentages for twice HPV31 and 51 DNA-positive women were significantly lower than those for HPV-free women.

## Discussion

In this report, the distribution trends of thrice, twice, and once positivity for HPV DNA of 12 HR-HPVs (16, 18, 31, 33, 35, 39, 45, 51, 52, 56, 58, and 59), two possibly carcinogenic genotypes (66 and 68), and two LR-HPV strains (6 and 11) were assessed in local older women. Our data shows that thrice and/or twice positivity for a given HPV DNA is highly prevalent in these subjects. For the total 1,020 person-time HPV infection, HPV52 and 58 were the top two strains, constituting 20.3 and 15.3% of person-time in total (thrice, twice, and once) and 14.5 and 12.2% of person-time for thrice positivity. When this trend was isolated by strain, thrice positive constitutions exceeded 50% for HPV58, 35, 52, 56, 18, 68, 31, 16, and 33; HPV58, 35, and 52 were the top three strains with >70% thrice positive ratios. The prevalence of once DNA positivity was relatively higher for the two LR-HPVs, 11 and 6. Regarding the age trend, the average age differed by strain in thrice, twice, or once HPV DNA-positive women. In general, the more positive test results women had, the older they were on average for HPV 66, 11, 16, 18, 51, 52, and 39. Although most HPV-infected and HPV-free women were NILM in cervical cytology, the percentages of women with thrice positivity for HPV52, 58, 16, 56, and 59 and women with twice positivity for HPV31 and 51 were significantly lower than the percentage in HPV-free controls.

Extrapolating from existing knowledge, once positivity for HPV DNA detected by a reliable approach, might mean a recent acquisition either as a new infection or reinfection, activation of a latent virus, and/or persistent infection (13). However, thrice or twice positivity for a given HPV DNA is more likely to indicate a persistent infection. Based on our results, the thrice and twice positive results for all HPV strains we detected, accounted for more than 60% of the person-time for each strain; in other words, most of the infections persisted for more than 2 years, and persistence lasting for 4 years was also common. However, as the upper limit is artificially controlled, we believe that longer infections are not uncommon.

Postmenopausal women form secondary population peaks of HPV infection due to senescence of cell-mediated immune control and possible new sexual partners (3, 14, 15). Our data showed a trend that the more positive results women had, the older they were on average for HPV 66, 11, 16, 18, 51, 52, and 39, which agrees with the age-related senescence of immunity being the main factor in promoting new infection or reinfection and activation of a latent virus, which makes persistent infection more likely to occur in older women.

Our previous report showed that 3.9, 7.6, and 5.1% of HPV-positive women displayed cervical cytology of ASC-US, LSIL, and HSIL, respectively. In this report, LSIL and HSIL were rare, and 95.2% of person-time were NILM. This result might benefit from a long-term and continuous screening program for cervical lesions in Shanghai. In addition, a study showed that older women with a long-term HPV infection had no cytological signs and a very small risk of subsequent cervical cancer (3, 14). Although we have not assessed the correlations between HPV infection and cervical histopathological changes, together with other reports, our data support the conclusion that reactivation from latent infection or persistent HPV infection in older women typically does not cause harm.

Reports have shown that HPV52, 16, and 58 were the top three most dominant HR-HPV genotypes prevalent in local areas, with positivity rates of 23.0 ~ 23.9%, 17.7 ~ 18.0%, and 16.9 ~ 15.7%, respectively (2, 16, 17). Our data indicate not only the ratios for person-time in total, but also that the thrice positive person-time of HPV52, 58, and 16 are still in the top three. In addition, twice and once positive ratios of HPV52 are higher. The twice and once positive ratios of HPV16 and 58 were higher but surpassed by those of the other genotypes.

A key uncertainty in the natural history of HPV infection is whether an HPV infection that becomes undetectable on repeat testing has truly cleared or whether the virus persists at low, undetectable levels (18). While no answer to this question can be derived by current science and technology, studies suggest that redetection of the same HPV genotype occurs in at least 10–20% of women who have been observed to have “cleared” the virus (18, 19), and multiple studies have confirmed immunologically controlled redetection or reactivation of a previously acquired type-specific HPV infection (18, 20).

## Data Availability Statement

All datasets generated for this study are included in the article/supplementary material.

## Ethics Statement

The studies involving human participants were reviewed and approved by the Review Board of the Ethics Committee of Medical Research at Minhang Hospital (Shanghai, China) approved the study protocol (Approval No. 2013SHMH004). The patients/participants provided their written informed consent to participate in this study.

## Author Contributions

LX and LJ: conception and design of study, revising the manuscript critically for important intellectual content, and drafting the manuscript. GH and JX: analysis and/or interpretation of data. All authors: approval of the version of the manuscript to be published and acquisition of data.

## Conflict of Interest

The authors declare that the research was conducted in the absence of any commercial or financial relationships that could be construed as a potential conflict of interest.
